# Incidentally Diagnosed Low-Grade Primary Peritoneal Serous Carcinoma Within the Umbilical Hernia Sac in a Male: A Report of an Extremely Rare Case and Review of the Literature

**DOI:** 10.7759/cureus.58534

**Published:** 2024-04-18

**Authors:** Samer Ganam, Ayesha Khan, Nicole Riddle, Joseph A Sujka, Christopher G DuCoin

**Affiliations:** 1 Surgery, Tampa General Hospital, Tampa, USA; 2 Gastrointestinal Surgery, University of South Florida Morsani College of Medicine, Tampa, USA; 3 Pathology and Cell Biology, University of South Florida Morsani College of Medicine, Tampa, USA; 4 Surgery, University of South Florida Morsani College of Medicine, Tampa, USA

**Keywords:** rare cancer, hyperthermic intraperitoneal chemotherapy (hipec), ppsc, primary peritoneal serous carcinoma, umbilical hernia sac

## Abstract

Primary peritoneal serous carcinoma (PPSC) is a rare tumor that develops in the peritoneum. PPSC originates from embryonic nests of Müllerian cells in the peritoneum, which are also present in the epithelium of the ovary. This similarity explains the histopathological resemblance between PPSC and low-grade serous ovarian carcinoma. While PPSC primarily affects women, it is an extremely rare occurrence in males, and it is believed that the significant difference in diagnosis rates between males and females is due to the inhibition of Müllerian system growth by substances produced by male Sertoli cells. These substances are present at higher levels in males, which may prevent the development of Müllerian system-derived tumors in men. We describe a 65-year-old male patient who presented for elective bariatric surgery and umbilical hernia repair, and an incidental finding of low-grade PPSC was made based on hernia sac pathology. The patient underwent further management, including tumor debulking and hyperthermic intraperitoneal chemotherapy (HIPEC), with positive outcomes. Long-term follow-up and oral letrozole treatment are planned.

## Introduction

Primary peritoneal serous carcinoma (PPSC) is an exceedingly rare epithelial tumor that originates in the peritoneum. The first documented case of PPSC was described in 1959 as a case report of mesothelioma of the peritoneum located in the pelvis [1]. Since then, researchers have put forth theories aiming to elucidate the origin of PPSC, one of which suggests that it arises from embryonic nests of Müllerian cells in the peritoneum. These nests are also found in the epithelium of the ovary, which may explain the histopathological similarities between PPSC and low-grade serous ovarian carcinoma [2]. PPSC occurs in women during the fifth and sixth decades of life, with an estimated incidence of 6.78 cases per million and a median overall survival of 21-23.5 months [3]. To date, only six cases of PPSC in men have been reported in the literature [4-9].

A possible explanation for the significant differences in PPSC diagnosis rates between males and females is that this tumor originates from cells implanted on the peritoneum, which may partly come from cells shed by the fallopian tube in females or secondarily the Müllerian system. Male Sertoli cells produce substances that inhibit the growth of the Müllerian system, starting around the seventh week of gestation. These substances reach their highest levels in early postnatal life, decrease during puberty due to the influence of testosterone, and then remain relatively stable throughout adulthood. These levels are consistently higher in males compared to females. This process may contribute to the absence of Müllerian system-derived tumors in males [10].

Patients diagnosed with PPSC present with general abdominal symptoms such as pain, bloating, nausea, vomiting, weight loss, and other unspecified issues [3]. Some risk factors have been described in the literature including increased age, obesity, hormone replacement, BRCA 1 and BRCA 2 mutation, and first-degree family history of ovarian cancer [3]. Other studies suggest that PPSC is not associated with BRCA mutation in men or women [10].

Gross features of PPSC may include omental cake, miliary studding, and discrete peritoneal masses. Microscopically, high-grade serous carcinoma displays a complex glandular and papillary pattern with significant nuclear atypia, while low-grade serous carcinoma demonstrates small papillae or solid nests with monotonous cytologic features.

The imaging modalities used for diagnosing and evaluating PPSC include CT, MRI, and PET-CT. The main treatment approach for PPSC involves surgery and chemotherapy, which typically includes primary debulking surgery combined with intraperitoneal or intravenous chemotherapy [3].

Here we present the seventh case in the literature of a male with PPSC, the third with low-grade type, and the first case of a male who was diagnosed incidentally during an elective surgery through an umbilical hernia sac, without any other suspicious finding or clinical symptoms.

## Case presentation

A 65-year-old male presented at the bariatric center for morbid obesity with a BMI of 48.82 kg/m² and the associated comorbidities type 2 diabetes mellitus (T2DM) treated with long-term insulin, primary hypertension (HTN), obstructive sleep apnea (OSA) managed with continuous positive airway pressure (CPAP), and coronary artery disease (CAD) with left circumflex artery stenting in July 2022. He had previously undergone gastric band surgery in April 2013, which was removed in July 2020. His previous attempts at weight loss had resulted in temporary success followed by weight regain. As a result, the patient was scheduled for an elective Roux-en-Y gastric bypass and an open repair of an incarcerated umbilical hernia.

Prior to surgery, he denied any abdominal pain, nausea, or diarrhea. His abdomen, including his non-reducible umbilical hernia, was non-tender to palpation. His review of systems was positive only for back pain secondary to sciatic nerve pain.

The abdomen was initially accessed through a supraumbilical incision with a 5-mm optical trocar. Adhesions were seen and adhesiolysis was performed to facilitate the placement of the trocars. No suspicious lesions were seen in the abdomen cavity or ascites. Subsequently, a Roux-en-Y gastric bypass surgery was performed. This was difficult due to the dense scar tissue caused by the patient's prior gastric band surgery and subsequent removal. Next, a wedge biopsy was performed to address fatty liver disease. After completing the bariatric surgery, the umbilical hernia was addressed using an open approach due to difficulties encountered in performing it robotically. An infra-umbilical incision was made, and a successful primary repair was completed. The hernia sac was resected and sent for pathology examination.

**Figure 1:**
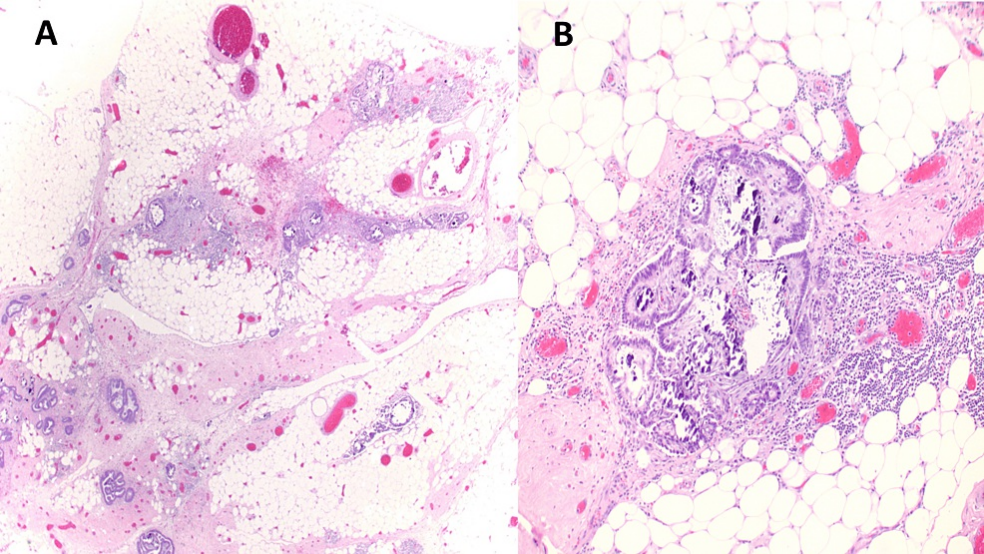
(A) Medium (20X) and (B) high-power (100X) images (H&E) showing infiltrating small nests and papillary structures with minimal nuclear atypia and scattered psammoma bodies.

The specimen grossly appeared as a 9.0 x 6.5 x 2.7 cm aggregate of yellow-tan, coarsely lobulated adipose tissue, some of which was covered by thin, tan fibrous tissue. Upon sectioning, the specimen showed adipose tissue that appeared slightly more fibrous in certain areas. No distinct lesions or areas of necrosis were observed. Histological analysis of the specimen revealed a papillary epithelial neoplasm with mild atypia and rare mitotic features, as well as scattered psammomatous calcifications (Figure 1). Immunohistochemistry showed the lesional cells to be positive results for CK7, PAX8, WT1, MOC-31, Ber-EP4, ER, and PR, while being negative for p16, p53 (wild type), CK20, calretinin, TTF1, and NKX3.1. The PHH3 staining highlighted rare mitotic figures up to 1/10 HPF, and the Ki-67 proliferation index varied up to 20-30%, which supports the diagnosis of primary peritoneal serous carcinoma, additionally, his CA-125 results were received later, and it appears to be elevated level of 110.9.

The liver biopsy revealed evolving cirrhosis with focal pericellular fibrosis and chronic steatohepatitis, characterized by macrovesicular steatosis affecting 30% of hepatocytes. There were no periodic acid-Schiff (PAS)-positive, diastase-resistant globules present, and there was no significant deposition of iron in the parenchymal or mesenchymal tissues. The patient had an uncomplicated postoperative hospital course with adequate pain control, stable vital signs, and clean incision wounds. He was discharged home in stable condition on postoperative day 1, with an outpatient follow-up appointment. 

Surgical Oncology, Medical Oncology, and Gynecologic Oncology were subsequently consulted. Surgical Oncology recommended an exploratory laparotomy for tumor debulking and staging, followed by hyperthermic intraperitoneal chemotherapy (HIPEC). Medical Oncology suggested chemotherapy after tumor staging and debulking, using a standard ovarian cancer protocol. Additionally, they planned to conduct Signatera molecular residual disease testing every three months to assess circulating tumor DNA. Gynecologic Oncology agreed with the plans for omentectomy, tumor debulking, surgical staging, and HIPEC, followed by chemotherapy. The patient was contacted by the oncologist and invited to the outpatient clinic to discuss the findings and the future treatment plan that was decided upon by all the disciplines involved.

Prior to the surgery, the patient underwent an abdominal MRI, which revealed mild ascites. Furthermore, a chest CT scan exhibited lymphadenopathy in the right anterior cardiac fat pad, along with a 2 cm subcarinal lymph node.

**Figure 2:**
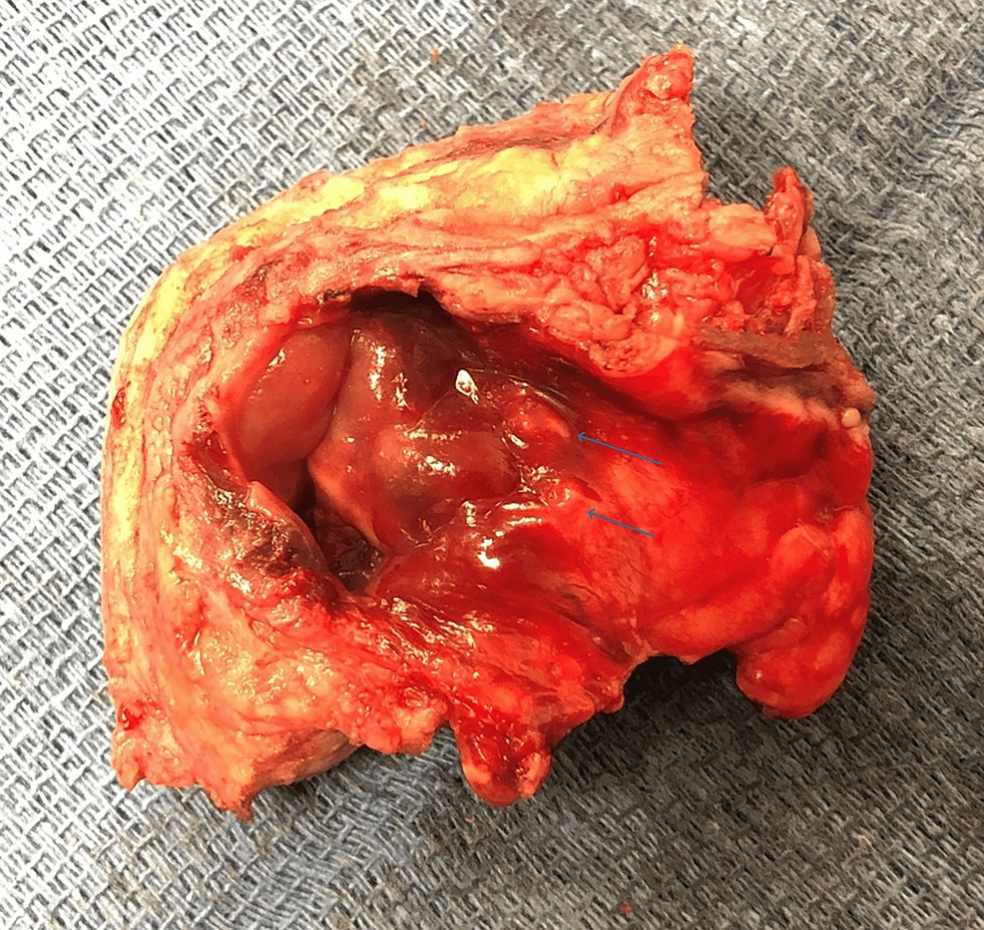
Two small carcinomatosis in the umbilical peritoneal wall.

**Figure 3:**
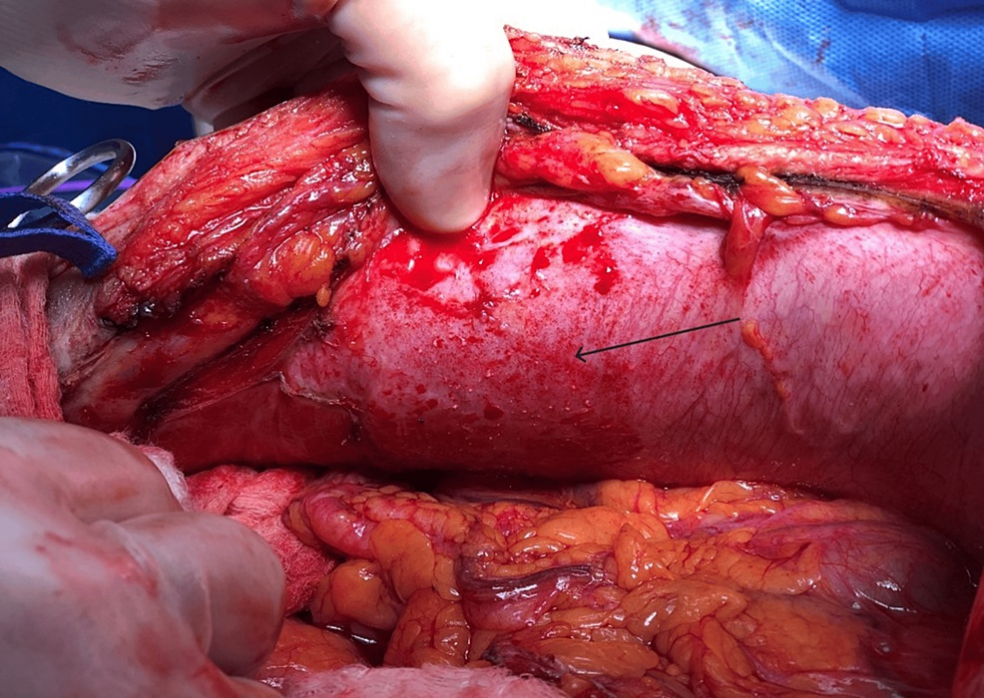
The miliary carcinomatosis in the right anterior abdominal wall.

The patient underwent an exploratory laparotomy approximately one month following the initial procedure for tumor debulking and HIPEC. A midline laparotomy incision was made to access the peritoneal cavity. Two small carcinomatosis lesions were observed in the umbilical peritoneal wall (Figure 2), along with omental disease. Additionally, small peritoneal implants were predominantly visualized on the right abdominal wall and right diaphragm, and no ascites were observed (Figure 3). 

**Figure 4:**
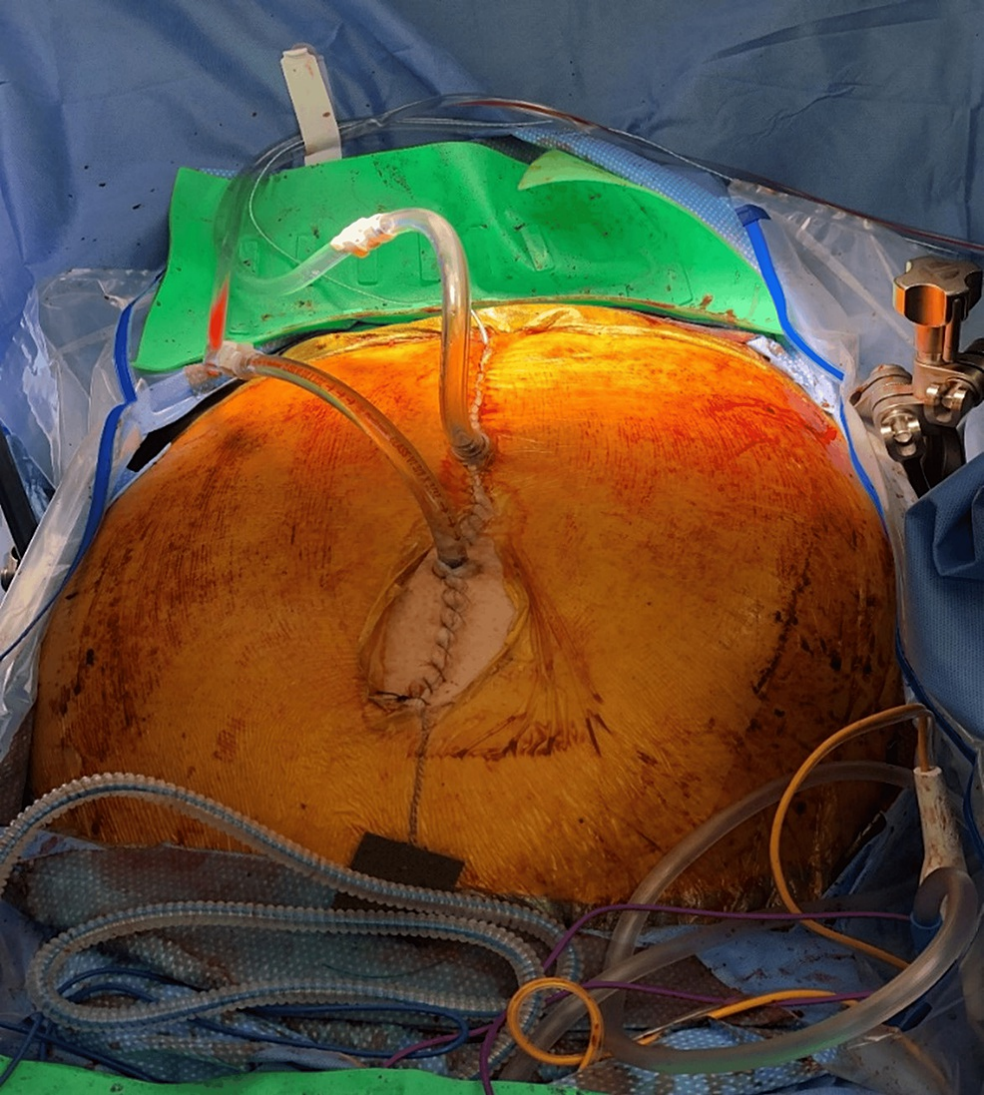
The patient is undergoing a 90-minute HIPEC treatment. Two tubes have been placed in the upper and lower abdominal cavities. HIPEC: hyperthermic intraperitoneal chemotherapy

A supracolic omentectomy was performed, with care taken to preserve the Roux limb in its antecolic position. The distal portion of the omentum was preserved as no disease implants were visualized. The omentum was resected on the left side and sent for permanent pathology. Subsequently, the right diaphragm was examined, and nodular areas were found on the right medial aspect of the diaphragm as well as the falciform ligament. The falciform ligament was excised, and the peritoneum of the right diaphragm was separated without causing any injury to the diaphragm. These specimens were sent for permanent pathology. There were also some capsular implants along the anterior aspect of the liver, which were removed. Additionally, a cholecystectomy was performed. An extensive peritonectomy of the right abdominal wall was then carried out, starting from the midline and extending to the paracolic gutter. Upon completion of the peritonectomy, hemostasis was achieved, and no additional tumor deposits were observed. The appendix was visualized and removed. The small bowel was examined for mesenteric deposits, which were identified and excised. Furthermore, a small implant on the rectum was also removed (Table 1). After achieving complete cytoreduction, the patient was connected to the HIPEC machine, which involved placing inflow and outflow catheters (Figure 4), followed by instilling 5 liters of fluid. Chemotherapy was administered once the output temperature reached 42 degrees. The patient underwent 90 minutes of HIPEC using carboplatin. The patient was continuously monitored to ensure appropriate urine output and a core temperature below 38.5 degrees. After completing the HIPEC, the abdomen was irrigated, and the catheters were removed. The incisions were closed once adequate hemostasis was achieved, and an incisional wound vac was placed.

**Table 1:** Pathological results from takeback exploratory laparotomy. HIPEC: hyperthermic intraperitoneal chemotherapy

Specimen	Results
Preperitoneal fat, excision	Suspicious for low-grade serous carcinoma
Urachus, Excision	Negative for malignancy
Omentum, Omentectomy	Multifocal low-grade serous carcinoma and psammoma bodies
Umbilicus, Skin excision	Low-grade serous carcinoma, psammoma bodies, and multinucleated giant cell reaction
Right diaphragm peritoneum, Excision	Focal low-grade serous carcinoma
Falciform ligament, Excision	Focal low-grade serous carcinoma
Gallbladder, Cholecystectomy	Negative for malignancy
Peritoneum, right abdominal wall, Peritonectomy	Low-grade serous carcinoma and psammoma bodies
Peritoneum, Right paracolic gutter, Peritonectomy	Focal low-grade serous carcinoma and psammoma bodies
Liver, Left liver capsule disease, Biopsy	Focal low-grade serous carcinoma and psammoma bodies
Liver, Liver nodule, Biopsy	Focal psammoma bodies, but no definitive carcinoma
Colon, Cecum mass, Excision	Fibrosis and focal psammoma body, but no definitive carcinoma
Appendix, Appendectomy	No definitive carcinoma
Umbilical nodule, Post HIPEC, Excision	Low-grade serous carcinoma, psammoma bodies, and foamy histiocytes
Small bowel adhesions, Post HIPEC, Excision	Psammoma bodies, but no definitive carcinoma

The patient was subsequently admitted to the intensive care unit for pain management and continuous monitoring. On the fourth day after the surgery, his diet was increased. He remained afebrile and did not require any antibiotics during the postoperative period. The surgical incision showed no signs of drainage or erythema, and the staples were appropriately in place prior to discharge. The patient was released on the fifth postoperative day.

As per the joint recommendation of Surgical and Gynecologic Oncology, the patient started chemotherapy with carboplatin (AUC 5), Taxol (175 mg/m^2^), and Avastin (10 mg/m^2^), followed by maintenance Avastin for six months to prevent the return of his disease. Per Gynecologic Oncology, further treatment would be considered with oral letrozole. At the five-month postoperative follow-up, the patient was feeling better with no evidence of disease recurrence. His recent CA-125 results came back normal, indicating positive progress. He will continue long-term follow-up to monitor his condition.

## Discussion

We presented a case of an asymptomatic patient with low-grade PPSC that was incidentally diagnosed during bariatric surgery and umbilical hernia repair. The diagnosis was made based on the pathological response from the hernia sac. Incidental diagnosis through the umbilical hernia sac has been described previously in a handful of female cases only [11]. So far, only six cases of symptomatic advanced PPSC in men have been reported in the literature [4,9].

In the previously reported cases of PPSC in males described in the literature, they presented with clinically advanced disease and exhibited symptoms such as abdominal pain, changes in bowel habits, weight loss, ascites, dyspnea, or chest pain due to pleural effusion. Physical examination showed reduced breath sounds and abdominal distention results from ascites. Our patient was incidentally diagnosed and denied experiencing any clinical symptoms that could be favorable for his prognosis, even though there is a lack of data on these specific asymptomatic male patients.

Immunohistochemically, both low- and high-grade tumors will typically demonstrate positivity for pancytokeratins, CK7, PAX8, BerEP4, and WT1, along with frequent estrogen receptor (ER) and progesterone receptor (PR) positivity. High-grade serous carcinoma shows abnormal p53 staining, diffuse and strong p16 staining, and elevated Ki67. In contrast, low-grade serous carcinoma exhibits wild-type p53 staining, negative/patchy p16 staining, and low Ki67 labeling. Furthermore, immunohistochemical evaluation is essential to differentiate from other pathologies, particularly gastrointestinal malignancies [3,12].

Our PPSC patient underwent immunohistochemical analysis, which showed positive results for CK7, PAX8, WT1, MOC-31, Ber-EP4, ER, and PR. However, it tested negative for p16, p53 (wild type), CK20, calretinin, TTF1, and NKX3.1. The PHH3 staining revealed rare mitotic figures, with up to 1/10 HPF, and the Ki-67 proliferation index ranged from 20-30%, further supporting the diagnosis.

Compared to the six previously published cases, this is the first case to test positive for MOC-31 and CD68. WT1 and a focal wild-type pattern for p53 were found to be positive in only one case before [8]. NKX3 (for bladder, prostate adenocarcinoma) and P16 were only tested in our case and were found to be negative. PR was performed in four out of six cases, with three cases showing negative results [5,6,8] and one case showing rare cells. In our case, PR was found to be positive (Table 2).

**Table 2:** Summary of the clinicopathological characteristics observed in all published cases of primary peritoneal serous carcinoma among male patients. CEA: carcinoembryonic antigen; ER: estrogen receptor; PR: progesterone receptor; EMA: epithelial membrane antigen; PSA: prostate specific antigen; NA: not applicable

	Current patient (2024)	Shah et al. (1998) [4]	Shmueli et al. (2001) [5]	Canbay et al. (2014) [6]	Xu et al. (2016) [7]	Makarenko et al. (2020) [8]	Guellil et al. (2022) [9]
Age	65	74	53	63	58	59	77
Histology	Low Grade	High-Grade	High-Grade	Not reported	Low Grade	Low-Grade	High-Grade
CA-125	+	+	+	+ (blood test)	NA	NA	NA
CEA	-	+	-	-	NA	-	NA
Stains							
CK7	+	+	+	+	+	+	+
PAX8	+	NA	NA	NA	+	+	NA
WT1	+	NA	NA	-	+	-	NA
MOC-31	+	NA	NA	NA	NA	NA	NA
Ber-EP4	+	+	NA	NA	NA	+	+
ER	+	NA	-	-	+	-	NA
PR	+	Rare cells	-	-	NA	-	NA
Focal wild-type pattern for p53	+	NA	NA	NA	-	+	NA
CD68	+	NA	NA	NA	NA	NA	NA
p16	-	NA	NA	NA	NA	NA	NA
p53 (wild type)	-	NA	NA	NA	-	NA	+
CK20	-	+	+	-	-	-	-
Calretinin	-	NA	NA	-	-	-	+
TTF1	-	NA	NA	NA	-	-	-
NKX3	-	NA	NA	NA	NA	NA	NA
D2-40	-	NA	NA	-	NA	-	NA
CK5/6	-	NA	NA	NA	-	-	NA
CAM5.2	NA	+	NA	NA	NA	NA	NA
EMA	NA	+	+	NA	NA	NA	+
B72.3	NA	-	NA	NA	NA	NA	NA
Vimentin	NA	-	-	NA	NA	NA	NA
Thyroglobulin	NA	-	NA	NA	-	-	NA
Chromogranin	NA	-	NA	NA	NA	NA	NA
Synaptophysin	NA	-	NA	NA	NA	NA	NA
HMWK	NA	NA	+	NA	NA	NA	NA
LMWK	NA	NA	+	NA	NA	NA	NA
CD15	NA	NA	+	NA	NA	NA	+
Mesothelin	NA	NA	NA	-	NA	NA	NA
CDX2	NA	NA	NA	-	NA	-	-
PSA	NA	NA	NA	NA	-	NA	NA
HBME	NA	NA	NA	NA	NA	NA	+
Desmine	NA	NA	NA	NA	NA	NA	-
IMP3	NA	NA	NA	NA	NA	+	NA

Regarding the tumor markers, CA-125 was detected to be positive in most cases, including our case, except in three cases where it was not tested [7-9]. Additionally, carcinoembryonic antigen (CEA) was mostly negative, except in one case where it was positive (Shah et al). All cases in the literature showed positive staining for CK7, and positive results were observed for PAX8, Ber-EP4, epithelial membrane antigen (EMA), and CD15 in all tested cases. Calretinin was found to be positive in only one out of five tested cases [9]. TTF1, CK5/6, D2-40, vimentin, thyroglobulin, and CDX2 were negative in all tested cases. CAM5.2 was tested in only one case [4], and it was found to be positive. B72.3, chromogranin, and synaptophysin were tested in only one case, and they were found to be negative [4]. HMWK and LMWK were tested in only one case [5], and they were found to be positive. Mesothelin, prostate specific antigen (PSA), and desmine were each tested once in three different cases, and they were found to be negative [6,7,9]. HBME and IMP3 were tested only once in two different cases and were found to be positive [8,9] (Table 2).

Considering the rarity of PPSC in men, the above findings are still not sufficient. More studies and cases with a comprehensive immunohistochemical work-up are needed to reach a consensus and draw conclusions, as well as to gain a better understanding of the molecular pathogenesis.

The main treatment for these patients should include cytoreduction surgery and chemotherapy, which means the removal of all macroscopic and visible implants [13]. According to the National Comprehensive Cancer Network (NCCN) guidelines, surgery, including debulking, should be combined with intraperitoneal or intravenous chemotherapy. In the previously described studies, only one patient received cytoreduction and debulking surgery accompanied by HIPEC, and that patient was still alive one year after the diagnosis [6]. The remaining cases, due to extensive and progressive disease, received either only HIPEC accompanied by intravenous chemotherapy or solely intravenous chemotherapy. Two cases died after three months and two months [4,5]. One patient died after one month [9]. Another patient died a few days later due to an unrelated cause (trauma) [7]. One patient developed pleural metastatic disease three months after the diagnosis, without additional information [8]. The last patient is still alive one year after his diagnosis [6]. Our patient underwent aggressive debulking surgery, which included omentectomy, peritonectomy, cholecystectomy, appendectomy, and removal of all macroscopic visible implants accompanied by HIPEC.

More male cases still need to be evaluated to understand this disease and establish the appropriate treatments and prognosis for these patients. However, based on the available evidence, it appears that a more aggressive treatment approach, including debulking and HIPEC, would be beneficial for patients.

## Conclusions

Aggressive treatment has the potential to improve prognosis and extend overall survival in PPSC patients. Furthermore, this is the first case that was incidentally diagnosed during elective surgery, specifically during the examination of the hernia sac pathology. It is important to understand that even in asymptomatic patients who undergo elective hernia repair, it is valuable to send the hernia sac for pathological examination. There should be a consensus among surgeons and other involved parties regarding this practice. More cases are still needed to gain a deeper understanding of the pathology, treatment types, and survival rates of male patients affected by this condition.
